# Federalism’s Fallacy at the Forefront of Public Health Law

**DOI:** 10.1017/jme.2023.26

**Published:** 2022

**Authors:** James G. Hodge, Summer Ghaith, Lauren Krumholz

**Affiliations:** 1: ARIZONA STATE UNIVERSITY, PHOENIX, AZ, USA

**Keywords:** Supreme Court, Constitution, Federalism, Rights, Liberty, Public Health

## Abstract

Amid undulating conceptions of the role and prowess of federalism emerges its central constitutional role: *protecting American liberties against unwarranted governmental intrusions*. To the extent that federalism is used as a guise for withdrawing fundamental rights to abortion by the U.S. Supreme Court in *Dobbs v. Jackson Women’s Health Organization*, individual rights are sacrificed in contravention of constitutional structural norms.

Many Americans understand federalism as the constitutional principle dividing powers between federal and state governments. The national government’s enumerated powers are distinct from sovereign powers reserved to the states via the Tenth Amendment.^1^ That’s federalism in a nutshell.^2^ Though easily conceptualized, constitutional jurisprudence over federalism is highly complex.^3^ Like a pendulum, federalism oscillates over time between federal and state powers, especially in the field of public health law where governments regularly clash over their authorities, as evinced vividly during the COVID-19 pandemic.^4^

Determining “who’s in charge” in public health emergencies and routine interventions,^5^ however, is not the sole determinant of federalism. Its constitutional role involves assessments of individual rights with mixed and sometimes notorious results.^6^ In its 1905 decision in *Jacobson v. Massachusetts*,^7^ the U.S. Supreme Court examined its own limits under federalism to balance state-based vaccine mandates against alleged individual liberty infringements amid a smallpox outbreak. Fast forward 117 years later, the Court invoked federalism for a very different end with immense public health repercussions. In *Dobbs v. Jackson Women’s Health Organization* (2022),^8^ it cast aside nearly 50 years of precedence to reverse a fundamental constitutional right to abortion in deference to states’ sovereign authorities.^9^ Essentially, the Court stripped individuals of constitutional protections partly out of respect for federalism.

Between these two seminal public health decisions lies a constitutional conundrum: *how exactly should federalism influence the outcome of rights-based assessments?* In each case, federalism is assessed to ultimately limit the scope or recognition of constitutional rights in different contexts. As examined below, this structurally-grounded view underscores a fallacy of federalism. The fabrication is not that constitutional rights may be limited by states’ compelling interests. No right is absolute.^10^ Rather, the misconception lies in how federalism is wielded to constrain individual rights. As espoused by Constitutional framers, scholars, and Supreme Court justices alike, federalism is about *protecting* Americans’ freedoms, not outright *denying* them.

## Federalism as a Stopgap to Judicial Interventions: *Jacobson*

In *Jacobson*, arguably the most famous and well-cited public health law case in American history,^11^ the Supreme Court considered purported liberty infringements under substantive due process raised by Reverend Henning Jacobson. Jacobson resisted a local mandate to be vaccinated for smallpox in Cambridge. His claims were ultimately rejected by the Court which recognized harm avoidance principles squarely built into constitutional rights to liberty.^12^ “[T]he liberty secured by the Constitution,” espoused Justice Harlan for the majority, “does not import an absolute right in each person to be, at all times and in all circumstances, wholly freed from restraint[s],”^13^ including vaccine mandates.

In *Dobbs*, however, the Court literally creates a public health crisis by derailing a firmly-held constitutional interest in furtherance of states’ sovereign powers. It simultaneously generates a legal crisis by provoking a wave of state anti-abortion laws, extensive litigation, and political wrangling.

In the Court’s view, individual liberty interests stop where direct harms to others may follow. Even as it affirmed its undeniable constitutional authority to adjudicate the meaning of “liberty,” the Court’s rights-based assessment was shaped by its recognition of prevailing principles of federalism prioritizing states’ roles in protecting the public’s health. Public health and safety “are matters that do not ordinarily concern the national government,”^14^ noted Justice Harlan. The Court “should not invade the domain of local authority except when it is plainly necessary to do so.”^15^ Justice Harlan acknowledged the Court’s limitations to counter legitimate exercises of public health powers by Massachusetts officials acting under their sovereign powers. In *Jacobson*, the crafted balance between individual liberty interests and state public health powers favors government not just because of principles of harm avoidance, but also out of respect for federalism.

## Federalism as a Factor for Limiting Constitutional Rights: *Dobbs*

The Supreme Court’s decision in *Jacobson* can be validated by the fact that liberty interests may be at their lowest ebb constitutionally in the face of deadly outbreaks, as seen as well during the COVID-19 pandemic. In *Dobbs*, however, the Court literally creates a public health crisis by derailing a firmly-held constitutional interest in furtherance of states’ sovereign powers. It simultaneously generates a legal crisis by provoking a wave of state anti-abortion laws, extensive litigation, and political wrangling.

In *Dobbs*, the Court resonates federalism concerns in its reliance on democratic processes and judicial neutrality to withdraw the long-standing constitutional right to abortion. Writing for the majority, Justice Alito pronounces from the onset how 26 states “have expressly asked this Court to … allow [them] to regulate or prohibit pre-viability abortions,”^16^ concluding “[i]t is time to … return the issue of abortion to the people’s elected representatives.”^17^ According to the *Dobbs* majority, when the federal constitutional right to abortion was originally bestowed in 1973 in *Roe v. Wade,* ^18^ 30 states prohibited abortion even as other states had liberalized their related laws. “*Roe* abruptly ended that political process,”^19^ observes Justice Alito. Concurring Justice Kavanaugh echoes the same need to “return” decision-making on abortion to states’ democratic processes, finding insufficient constitutional authority for the Court to create new rights.^20^ The majority argues further how abortion is neither deeply-rooted in the nation’s history nor was it outside the realm of states’ criminalization when the Fourteenth Amendment was ratified in 1868.^21^ As a result, the *Dobbs* Court characterizes precedence in *Roe* and *Planned Parenthood v. Casey* ^22^ as “substantial restrictions” on the inherent authority of states to regulate abortion.^23^

In essence, federalism won out in *Dobbs* over continued recognition of constitutionally-recognized rights. On the day *Dobbs* was released, June 24, 2022, Senator Rick Scott (R-FL) celebrated the decision for “defend[ing] … the foundational principle of federalism.”^24^ Senator Kevin Cramer (R-ND) declared *Dobbs* a win for “states’ rights.”^25^ One commentator analyzed how *Dobbs* weaponized states’ rights,^26^ rekindling “harsh images of federalism”^27^ from prior decades buttressing state resistance to desegregation and other civil rights that are now firmly recognized at every level of government.^28^

## Federalism as a Preserver of Rights and Freedoms

Supreme Court deference to state sovereignty in reversing a firmly-held constitutional right to abortion in *Dobbs* misconstrues underlying foundations of federalism. Ultimately, federalism is not about denying inherent liberty interests under substantive due process; *it is about promoting them*. The Constitutional framers clearly intended federalism to protect the “liberty of individualized citizens,”^29^ by offering “double security” for “the rights of the people.”^30^ In the Federalist papers, Alexander Hamilton explained how “federalism is a safeguard … against the overextension of government’s power.”^31^ As one modern commentator espouses, federalism provides a “two-tiered protection of individual rights” through the Fourteenth Amendment affording a “guaranteed minimum of protection,” with states able to proffer greater assurances.^32^

Multiple constitutional law commentators conclude how adjudicating federalism invariably entails promotion of individual rights. Professor Jonathan Adler equates federalism directly with the protection of individual rights.^33^ Dean Erwin Chemerinsky argues how it enhances liberties in furthering societal objectives.^34^ In the context of civil rights, Professor James Blumstein illustrates how federalism “decentralizes decision-making to promote autonomy, democracy, and freedom.”^35^ Another commentator surmises, “federalism is not merely a means to diffuse power; it is a principle to … [protect] the rights and privileges of all citizens.”^36^

In 2002, Michigan Supreme Court Chief Justice Maura Corrigan described the “integral role” of the judiciary in protecting federalism to safeguard individual rights.^37^ Her view is backed by existing U.S. Supreme Court jurisprudence prior to *Dobbs*.^38^ In *Boyd v. United States* ^39^ (1886), the Court rejected criminal charges against an individual compelled to produce private documents contrary to Fourth Amendment privacy protections. Justice Bradley proclaimed the Court’s duty is to be “watchful for the constitutional rights of the citizen … against any stealthy encroachments.”^40^ A century later in *Garcia v. San Antonio Metropolitan Transit Authority* ^41^ (1985), dissenting Justice Powell observed how “the constitutionally mandated balance of power [is] … designed to protect our fundamental liberties.”^42^

Numerous Justices have since explicitly observed how federalism functions to preserve individual rights in decisions (among others) related to (1) retirement requirements for state judges;^43^ (2) gun possession;^44^ (3) prescribing rights for physician-assisted suicide;^45^ (4) alleged possession or use of a chemical weapon;^46^ and (5) licensing of sports gambling.^47^ Collectively these decisions support how federalism “was adopted by the Framers to ensure the protection of ‘our fundamental liberties,’”^48^ which are distinct from and “not simply derivative of the rights of the States.”^49^ As Justice Kennedy concludes in *Bond v. United States* in 2011, “[f]ederalism secures the freedom of the individual.”^50^

That federalism is about protecting, promoting, and even advancing liberty interests intimates how the *Dobbs* Court erred in concluding that states’ interests warrant a return of regulatory authority over abortion. To the contrary, Americans’ fundamental freedoms adjudicated by Justices nearly a half-century ago merit continued respect for bestowed rights against their summary withdrawal. Under *Dobbs’* majority reasoning, manifold other liberty interests previously framed by the Court (e.g., contraception, sexual intimacy, marital equality) may be at risk of reversal with epic potential impacts on population health and well-being.^51^ Doing so under the guise of federalism not only disrespects Americans’ freedoms, but also resounds historically-rejected premises of states’ rights as constitutionally-viable reasons to deny individual liberties. Positing constitutional structural principles like federalism in support of reversals of settled, rights-based reasoning is a dangerous path for the Court to follow. Ultimately, it may find the trail ends where federalism begins: *at the doorstep of liberty*.

## Note

The authors do not have any conflicts of interest to report. No specific entity provided funding for the production of the manuscript.
